# Depression and Obesity in Patients With Psoriasis and Psoriatic Arthritis: Is IL-17-Mediated Immune Dysregulation the Connecting Link?

**DOI:** 10.3389/fimmu.2021.699848

**Published:** 2021-07-21

**Authors:** Efterpi Zafiriou, Athina I. Daponte, Vasileios Siokas, Christina Tsigalou, Efthymios Dardiotis, Dimitrios P. Bogdanos

**Affiliations:** ^1^ Academic Department of Dermatology, University General Hospital of Larissa and Faculty of Medicine, School of Health Sciences, University of Thessaly, Thessaly, Greece; ^2^ Academic Department of Neurology, University General Hospital of Larissa, Faculty of Medicine, School of Health Sciences, University of Thessaly, Thessaly, Greece; ^3^ Department of Medicine, Democritus University of Thrace, Alexandroupolis, Greece

**Keywords:** depression, IL-17, IL-23, immunity, obesity, psoriasis, psoriatic disease

## Abstract

Patients with psoriasis are frequently obese and experience anxiety or suffer from depressive disorders. The immunopathogenesis of psoriasis and indeed psoriatic arthritis is largely based on the pivotal role of IL-17/IL-23 axis, to an extent that currently monoclonal antibodies selectively inhibiting IL-17 or IL-23 are routinely used for the treatment of psoriatic diseases. Emerging data, demonstrating a decisive role for IL-17 and IL-17 producing cell subsets, such as Th17 in the induction and progression of obesity and depression has led authors to suggest that psoriatic disease, obesity and anxiety/depression may indeed be interconnected manifestation of a state of immunedysregulation, the linked being IL-17 and its related cells. We discuss this hypothetical link in depth taking into account the beneficial effects anti-IL17 and anti-IL-17 receptor inhibitors in treating psoriatic disease and the on-going debate as to whether these biologics may exert a direct or indirect effect in ameliorating concomitant obesity and depressive disorders, which are frequently noted in the same patient.

## Introduction

Depression is a frequent comorbidity of various autoimmune rheumatic and immune-mediated diseases, including psoriasis (Ps) and psoriatic arthritis (PsA) (1). More then a third of patients with these diseases experience anxiety, depression, and even suicidal ideation and behaviors (SIB), which is not always immediately relevant with the activity of the underlying disease (2–4). In fact, the rate of depression can be higher in patients with PsA than in those with Ps (5). The cause–effect bond between depression and Ps (as well as PsA) is puzzling (6). Remission of the disease, following successful treatment, especially with biologics has a positive effect on depression and anxiety, but not always. Chronic inflammation has been considered a favorable trigger of depression and chronic medical illness (7). The immunobiological basis of depression’s development has started to emerge following studies demonstrating a fine imbalance between pro-inflammatory and anti-inflammatory cytokines (8–10). IL-17 has emerged as a master cytokine for the immunopathogenesis of psoriatic disease, leading to the development of the plethora of agents specifically inhibiting its deleterious effect (11–15).

The direct or indirect effect of such biologics in fighting depression in patients with immune-mediated or autoimmune diseases such as Ps and PsA remains elusive (16). The lack of information resulting from inconsequential clinical data will become less elusive in the coming years as monoclonal antibodies (mAbs), such as those specifically inhibiting IL-17 and IL-17 receptors, are currently approved treatments for psoriatic disease patients and data are continuously analyzed and published (17). The impact of mAbs specifically inhibiting that; cytokine in depression, anxiety and even suicidal ideation and behaviors (SIB) will also become more evident, as animal models of depression become more sophisticated resembling more and more the human disease. The question arising from the clinical use of the approved drugs is whether anti-IL17 treatment is efficacious in combating depression, irrespectively of the disease patients are suffering from (Ps, PsA or other spondylarthropathies) and whether the magnitude of IL-17 inhibition can predict the performance as per depression scale.

So far, it has become apparent that patients suffering from depression have elevated levels of circulating IL-17 in their serum and that the percentage of Th17 cells, the T cell predominantly producing this cytokine, are also increased in people with depressive disorders (18). What is not clear and remains a matter of debate is whether this increase is an epiphenomenon resulting from the disease or whether the increase *per se* is playing a decisive role in the development and progression of neuroimmune depressive disorders in isolation or in combination with other non-immunologically relevant mechanisms (19). Research clinical trials using IL-17 or IL-17R blockers for the treatment of depressive disorders have not been initiated so far. It is worthy to mention data from two clinical trials, based on biologic therapy, and in particular that using infliximab which is a chimeric IgG1 mAb that blocks TNF-α for the treatment of refractory depression (20, 21). None of the two achieved its primary outcome, and infliximab did not appear to reduce symptoms of depression compared to placebo, though a favourable outcome has been reported in those patients with inflammatory indices (21).

It has also been well documented that people with depression are more frequently obese compared to those without and that obese people, because of their internal and external stigma, are experiencing more frequent depression. Again, it is not clear what is the pathophysiologal impact of IL-17 in the direct induction of obesity. A provocative hypothesis is taking into consideration that an impairment of immunoregulatory mechanisms, characterized by the functional inability of the immune system to promote the expression and production of IL-17 and other suppressory cytokines, is directly linked to the overexpression of IL-17 for cellular subsets such as—but not limited to—Th17.

If IL-17 is critical for the immunedysregulation noted in patients with depression, it should be expected that patients with that disorder at early stages of the disease will have well documented increased expression of IL-17, and that over time, serum IL-17 levels as well as the levels of Th17 and other IL-17-producing cells will become significantly amplified, especially in those patients who become more depressed and more obese because of the underlying disease. Prospective studies reporting on that are currently missing, but experimental data in animal models are rather informative and provide a wealth of information, which can lead to a better understanding of the complex nature and close interplay between immunedysregulation in psoriatic disease and its impact on depression and obesity.

We and others have shown that is IL-17 per se, as well as IL-17 axis, is pivotal for the development and progression of Ps and PsA (12, 13, 22–27). More recently, the imperative role of Il-17 in the induction and maintenance of ankylosing spondylitis and other spondylarthropathies has been revealed (28).

## IL-17 Selective Biologics for The Treatment of PS and PSA

Four biologic therapies targeting either IL-17 or IL-17R have been approved for the treatment of Ps. Secukinumab, a fully human IgG1κ mAb (29) and ixekizumab, a humanized IgG4 mAb (30, 31) selectively bind and neutralize IL-17A. Brodalumab is a fully human IgG2 mAb that binds and inactivates the IL-17A receptor leading to the inhibition of either IL-17A, IL-17C, IL-17E and IL-17F (32). Finally bimekizumab, a humanized IgG1 mAb, which selectively neutralizes IL-17A and IL-17F, is approved for the treatment of Ps but is also efficacious in PsA (33).

## IL-17 Levels and TH17 Cell Subsets In Patients With Depression

The neurological and psychiatric disease related implication of Th17 and Il-17 mediated cell damage is the focus of intense research and has just been started emerging. Th17 cells have been considered likely inducer of brain damage (34). Th17 induce neuronal cell death and promote neuronal toxicity in experimental autoimmune encephalomyelitis, the animal model of multiple sclerosis (35) and IL-17 mRNA is overexpressed in active MS brain lesions (36), while IL-17 production from central nervous system resident T lymphocytes and glial cells are associated with disease-activity (37). CD8+ T cells producing IL-17 are elevated during disease-relapses compared to disease-remission (38). Work in mice has clear demonstrated that though not directly, IL-17 plays an important role in MS, as mice deficient for IL-17A/F escape from disease’s appearance. Immunome data demonstrate that paediatric patients with drug refractory epilepsy are characterized by an IL-17 inducing CD4 and CD8 cell subset profile, which likely contributes to epileptogenesis (39) while autism spectrum disorders are characterized by an imbalance between proinflammatory Th17 and suppressory T regulatory cells (Tregs) (40). Th17 cells are increased in patients with stable schizophrenia (41).

Depression is associated with the elevation of proinflammatory cytokines among which IL-17 appears to be one of those found elevated in patients with major depressive disorders (42, 43). Accumulated evidence suggest that the cytokine milieu noted in patients with depression, as well as that well-characterized in animal models of depressive disorders, underlined the important role of IL-1β, TNF-α and IL-6 (44–46). A recent meta-analysis investigating children and adolescents with depression has found that IL-6 predicts the future development of depression and conversely that the establishment of depression is a significant predictor of IL-6 increase (47). That effect appears to be influenced by other factors like gender or stressful life events. A longitudinal cross-lagged twin study has provided compelling evidence supporting the imperative role of IL-6 increase as an independent risk factor of depression rather than an epiphenomenal consequence of disease’s establishment (48). In animal models of depression, IL-6 (and/or IL-1β appears to be instrumental for the development of chronic stress and depression-like behaviors (49). Also, numerous studies have shown that patients with major depression have elevated levels of TNF-α and that fluctuation of this pro-inflammatory cytokine (as well as that of IL-6) influences the mood behavior of the affected individuals (for review see (50). Furthermore, some data suggest that antipsychotic drugs exert an anti-inflammatory effect and decrease TNF-α and IL-6 levels in murine models of the disease (51). Intriguingly, however, a recent systematic review, meta-analysis and meta-regression including 38 eligible studies representing 58,256 failed to identify a prospective association of depression with TNF-α and a small association with IL-6 (45). Also, as we mentioned previously, data from a limited number of clinical trials have failed to identify a beneficial effect of anti-TNF-α biologics in patients with mood disorders (20, 21), while such drugs are successfully used for the treatment of psoriatic disease, other autoimmune rheumatic diseases and several immune-mediated inflammatory diseases.

Experimental work has been redirected to other newly identified pro-inflammatory cytokines, including IL-17. IL-17A mediated disruption of the blood-brain barrier by Th17 cells is well documented, and treatment of mice with IL-17A neutralizing antibodies prevents such a disruption (52). Though limited, data from studies in patients with depressive disorders are also noteworthy (**Table 1**). Interestingly, a serological study has found higher IL-17 levels in blood samples in 41 patients with major depressive disorder compared to those noted in 40 healthy age-matched controls with no history of malignancies or autoimmune diseases (53). However, lack of an association between blood levels of IL-17A and depression has also been reported, as anti-depressants appear not to exert an effect on IL-17 levels (57). The levels of IL-17 gene expression among 190 patients with depression were higher compared to 100 healthy individuals, while the mean mRNA expression of the immunoregulatory Foxp3 was considerably reduced in patients suffering from depressive disorders compared to the control group (54). A study in 40 patients with major depressive disorders and 30 healthy controls has found an imbalance of Th17/Treg ratio compared to healthy controls documented by a significant increase in peripheral blood mononuclear cell Th17 cell numbers, and a decrease in T regulatory cells (55). The same study also reported higher mRNA expression levels of retinoic acid-related orphan receptor-γt (RORγt), which is the specific transcription factor of Th17 cell and increased IL-17 serum levels in depressed patients compared to healthy controls (55). Another study found that women with major depressive disorder have increased Th17 and increased serum IL-17 compared to controls (56).

**Table 1:** A summary of human studies reporting on the role of IL-17 in depression.

Type of Study	Major findings	Comments	Reference
Serological study in patients with major depressive disorder	Patients with major depressive disorder have higher serum IL-17 compared to those found in age-matched healthy controls	The number of samples was limited to reach safely conclusions.	(53)
Molecular study in patients with depression	Cumulative mRNA levels of IL-17 gene expression in peripheral blood samples were significantly higher in 190 patients with depression compared to 100 healthy individuals	Sample size was sufficient enough but this study was not prospective and the effect of anti-depressants was not evaluated	(54)
Immunocellular study in patients with major depressive disorders & Serological study	Th17/Treg ratio of peripheral blood mononuclear cells was increased in 40 patients with major depressive disorders compared to 30 healthy controlsSerum levels of IL-17 measured by ELISA are increased in patients with major depressive disorders compared to 30 healthy controls	Interesting study showing an increase of Th17 cells and a decrease of Treg cell subsets	(55)
Cellular and serological study in patients with depressive disorder	Percentages of Th17 cells and serum levels of circulating IL-17 were increased in 30 patients with major depressive disorder compared to 30 sex-, age-, body mass index, ethnicity- and smoking status-matched healthy controls	The study included BMI, smoking-status and demographically matched healthy controls, which make the data more reliable.As for the previous studies the study in not prospective and the kinetics of cell subsets and circulating levels of IL-17 are not available	(56)

**Figure 1:**
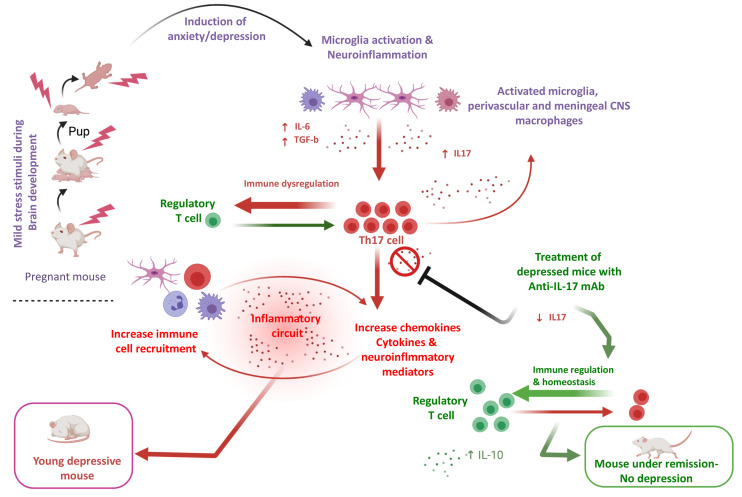
An-IL-17-mediated hypothesis of depressive disorder in an experimental model. In a young adult depression mouse model exposed to cumulative mild stress (CPMS) characterized by microglial activation, IL-17 in brain and blood, as well Th-17 cells are elevated. A hypothesis based on the assumption that microglia activation is pivotal for the increase of pro-inflammatory cytokines such as IL-16 and TGF-b, which polarize CD4+ T cells towards Th-17 is formulated. Experimental data suggest that anti-IL-17 mAb treatment, diminishes IL-17 induction and Th-17 differentiation and ameliorates anxiety and depression-like behaviors (61) (prepared with BioRender).

Considerable evidence in mice supports the notion that Th17 cells endorse susceptibility to depression-like behaviors (58). The percentage of Th17 cells is significantly increased (up to a 3-fold) in the brain of mice demonstrating depressive-like behavior compared to mice that do not have such behavior (59). Of interest, at the experimental level oral administration of BALB/C to mice sensitized by ovalbumin with the tricyclic-antidepressant desipramine diminished symptoms of allergic rhinitis symptoms in mice, up regulated CD4+CD25+Foxp3+ Treg cells and reduced CD4+IL-17+ Th17 cells, which were significantly increased in mice not receiving this antidepressant. Finally, *in vitro* addition of serotonin and treatment of MDD patients with selective serotonin reuptake inhibitors (SSRIs) reduced the production of Th17/Tc17-related cytokines by CD4+= and CD8+ T cells (60). Treatment of depressed mice with anti-IL-17 mAb inhibits glial differentiation and ameliorates anxiety and depression behaviors (61). In the human setting, CD4+CD25+ Tregs are found decreased in patients with major depression (62) (**Figure 1**).

### IL-17 and Depression in Psoriatic Disease

A population-based cohort study found that patients with Ps who have elevated levels of IL-17A also have increased risk for depression and anxiety disorders. In a murine imiquimod model of psoriatic disease, administration of IL-17A was associated with acceleration of depression-like symptoms, while treatment with an anti-IL17A antibody diminished depression-like symptomatology (63). A recent case-based study reported three patients with moderate-to-severe Ps and comorbid depression, who were successfully treated with brodalumab (PASI 100 after treatment with brodalumab); in two of those, depressive symptoms either improved or resolved suggesting that the use of brodalumab is able to improve both skin lesions and depression (64). These results rather contradict those suggesting a potential link between SIB and brodalumab use, as there are data from the clinical Phase 3 Studies (AMAGINE-2) which report three events of suicide attempt that occurred in only one individual out of 486 participants (Patient-yr = 379.7) who received a constant dose of brodalumab 210 mg Q2W (65), which makes dermatologists rather hesitant to prescribe that mAb (65, 66), despite the lack of concrete causality (67). The exact mechanism by which brodalumab may indeed exert or participates in suicidal behavior has not been studied in the affected individual or in any other setting. The distinctive mechanism of action of brodalumab involves the broader blockade of IL-17 isoforms binding IL-17RA. If this action can provide a mechanistical explanation of the assumed suicidal ideation induction remains elusive. Studies of the effect of anti-IL17 RA or other anti-IL17 neutralizing antibodies in animal models of suicidal behavior (68) may shed a light on this provocative topic.

### Depression and Suicide Ideation Following Anti-IL17 mAb Treatments in Ps and PsA

A recent two-year US pharmacovigilance report on brodalumab usage collecting data from 2,677 patients with an estimated exposure of 1,656 patient-years reported just 25 reports of depression but no suicide attempts (69). Data on the safety of ixekizumab in adult patients with plaque Ps, PsA and axial spondylarthritis from 21 clinical trials of 8,228 patients with an ixekizumab exposure of 20,895.9 patient-years have reported depression during ixekizumab treatment occurred in 203 patients with Ps, 37 patients with PsA and 13 patients with axial spondylarthritis. The cumulative data over the total exposure period have shown incidence rates of reported depression to be low (≤2.2 per 100 PY across indications) and decreased across the treatment periods. Of importance, the same study found that suicidal behavior/self-injury was present in 17 patients with Ps, one patient with PsA and two patients with axial spondylarthritis (70). A very recent *post hoc* Analysis of the Italian SUPREME multi-centre (50 sites) study has found that anxiety was resolved in 67 and 71% of Ps patients at weeks 16 and 48, respectively and that depression symptoms were improved in 81.3 and 70.6% of patients at weeks 16 and 48, respectively (71).

### IL-17, Obesity and Depression in Psoriatic Disease

Currently, it is projected that by 2030, 20% of the world’s population will be obese and 38% will be overweight (72). New piece of evidence suggests that the spatial prevalence of comorbid obesity/depression is not a random phenomenon and indeed common denominators at the cellular and immunophysiological level may account for mutual interactions between obesity and depression (73). Obesity is related to a higher grade of inflammation and this may hold true for depression too. Metabolic manifestations seen in obese people such as cardiovascular diseases and diabetes have been attributed at least in part in obesity-related chronic inflammation stemming from adipose tissue (74). Adipose tissue induces IL-6, which is an important mediator for CD4+ T cell polarization to Th17 cells (75). Blood concentrations of IL-17 (as well as IL-23) appear to be elevated in obese (BMI: 30–48 kg/m^2^) compared to slim women (BMI: 18–25 kg/m^2^) (76) (**Table 2**).

**Table 2:** A summary of representative studies reporting on the role of IL-17 in obesity.

Type of Study	Major findings	Comments	Reference
Serological study in obese women	Circulated serum levels of IL-17 (as well as IL-23) were elevated in 20 obese women obese women compared to 20 lean women	Sample size is too small	(76)
Serological study in obese men and women	Plasma IL-17 levels were higher in 42 volunteers with a BMI >35 compared to those of 34 volunteers with normal BMIs. IL-17 levels were significantly higher in men with a BMI >35 than women with a BMI >35. IL-17 was elevated in those with a BMI >35 that had type 2 diabetes versus those without type 2 diabetes	Relatively small size study limited to plasma sample tests	(77)
Serological study in women with psoriasis and metabolic syndrome	IL-17 levels were higher in women with psoriasis and metabolic syndrome compared to those without	Small cohort tested	(78)
Serological study in patients with metabolic syndrome undergoing nonlinear resistance training	No association was found between IL-17 levels and metabolic syndrome variables and levels of IL-17A were not affected by training	Main cohort and control group small in size (in total 22 individuals)	(79)
Serological study in obese patients undergoing bariatric surgery	Plasma levels of IL-17A in 18 patients significantly decreased 6 months post-operatively	Small size tested	(80)
Serological study in obese and non-obese women participating in a randomized double blind placebo controlled trial investigating the effect of vitamin A	The mean concentration of circulating IL-17 was decreased after vitamin A supplementation in obese as well as non-obese women	One of the very first studies to demonstrate that vitamin A has an effect on IL-17 levels in obese women	(81)

**Figure 2:**
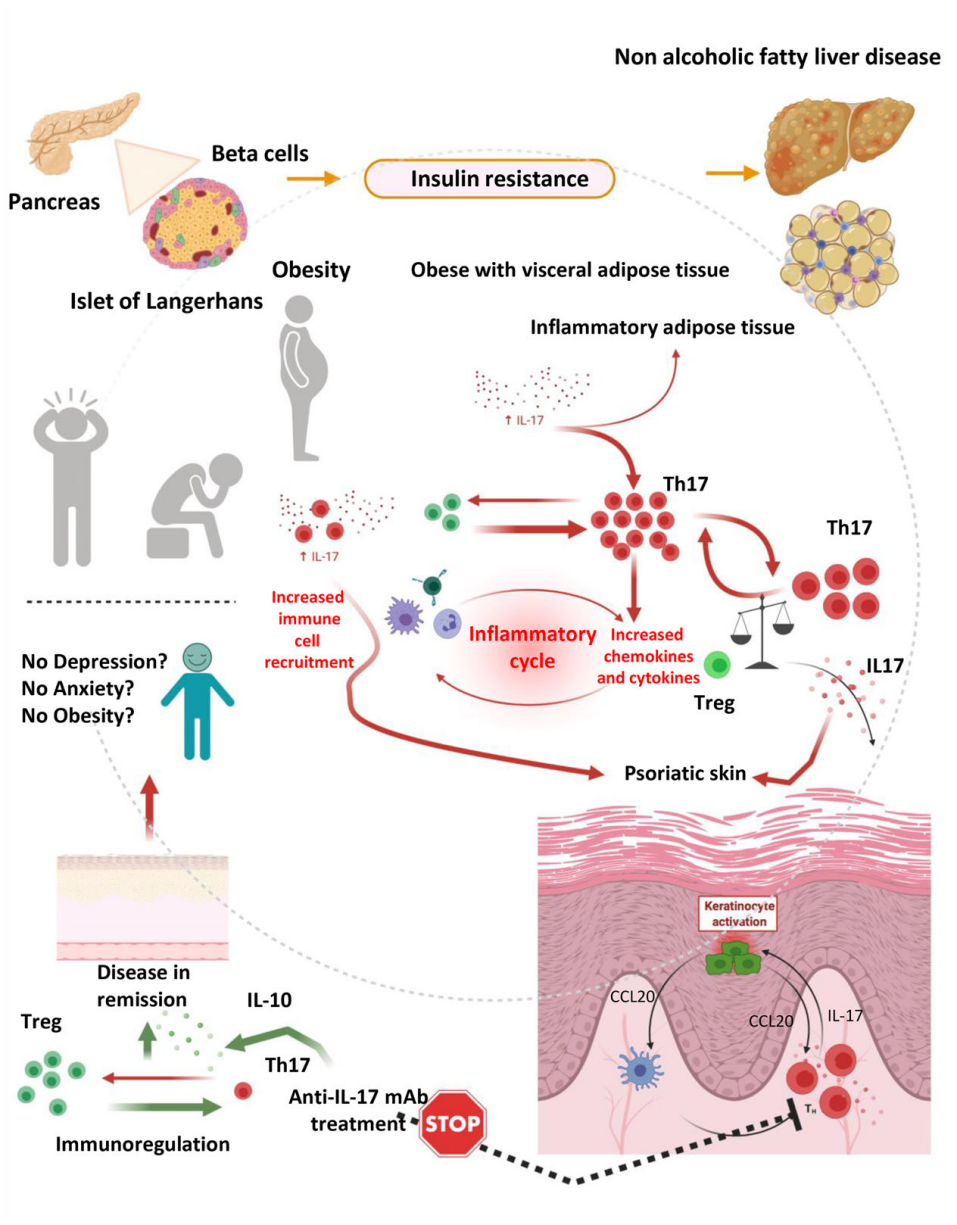
The hypothesis of immunedysregulation in obese depressed patients with psoriatic disease. An inflammatory response mediated by IL-17 producing cells such as Th17 is playing a pivotal role in the development of obesity, depression and psoriasis, in isolation or in combination. Immundysregulation manifested as increased expression of IL-17 and IL-17 producing cell subsets and diminished expression of regulatory cells (Tregs) and related cytokines (such as IL-10) is driving complex reactions leading to the development and progression of psoriatic disease and concomitant anxiety, depression and obesity. Those manifestations can partially be attributed to the inflammatory milieu, which fosters consistent inflammation, keratinocyte activation, cellular damage and skin destruction, as well as interconnected depressive disorders and weight gain. This vicious circle is stopped by IL-17 selective inhibitors, which can lead to the remission of skin lesions (and in the remission of arthritogenic features in the case of psoriatic arthritis or axial spondylarthritis) (prepared with BioRender).

Of relevance, Ps and obesity are interconnected on the basis of various common denominators (82). For example, significant weight loss can improve psoriatic lesions and attributed to disease-remission. In addition, obesity is associated with higher incidence and prevalence of Ps. Finally, work in animals has shown that BMI increase participates in the development of Ps, while clinical studies have shown that increase in body weight participate in the relapse of psoriatic lesions and/or the *de novo* appearance of the disease (82). On the basis of that it becomes apparent that Ps, obesity and depression are interlinked and that IL-17, which significantly contributes to disease development may account for the induction and continuation of all three denominators in the same patient (**Figure 2**).

Intriguingly, recent data has shown that ixekizumab was efficacious in the treatment of moderate-to-severe Ps irrespectively of body weight (83). In most research clinical trials, anti-IL-17 inhibitors did not exert any effect on body weight increase. In AMAGINE 1, a phase 3 trial of brodalumab, treatment with this mAb showed higher rates of disease skin improvement (PASI 75 and PASI 90) at weeks 12 and 52 in normal weight patients compared to that noted in obese psoriatic patients (66, 84). Notably, IL-17 inhibitors are very effective independently from body weight; however, they tend to present better clearance rates in normal weight patients.

However, a recent study failed no correlation between body mass index and IL-17 expression in 95 patients with depression (85) and secukinumab-induced skin remission in patients with Ps does not reduce body weight after 12 or 24 weeks of treatment (86). A phase 4 randomized, multi-centre, open label, parallel group, active comparator-controlled study with a duration of 28 weeks and a 28 week extension phase (MEDABOLYX, NCT03440736) recruits patients in an attempt to assess whether secukinumab with lifestyle intervention can improve both skin symptomatology and cardiometabolic status compared to secukinumab alone. Though, the design of the study is on immediately addressing the emerging issue as to whether the IL-17 blocker can improve body weight/BMI, the results of the study could be informative and are highly anticipated.

Several studies, including those conducted by our group, have revealed a negative correlation between IL-17-producing cells and IL-10-producing or other regulatory cell subsets in patients with Ps and PsA, but it is not clear whether these negative correlations are largely depicted in patients stratified in accordance to the presence of obesity (or depression) (22, 87, 88). Of relevance, secukinumab appears to exert an intriguing obliterating effect on disease-related autoantibodies in patients with Ps and PsA (89).

In the last decades, extensive piece of information underline that cutaneous Ps and PsA patients are at higher risk of developing obesity and cardiovascular disease. A major player for that increase, particularly in the industrialized countries, has been attributed to the adaptation of a western lifestyle with less physical activity and diet with high fat and carbohydrate, as well as excessive sodium consumption favor the development, all of which contribute to overweight and obesity (90). Depression is also a comorbidity, which patients with psoriatic disease frequently need to deal with. Whether these comorbidities contribute *via* an immunobiologically sound mechanism in disease development, relapses or progression or they are just epiphenomena, is a matter of heated debate. Needless to mention, that a significant impact on the resolution or improvement of anxiety and depression related symptoms, as well as on controlling weight, it is likely due to the positive effect the disease remission may exert in patients with Ps and PsA.

## Conclusion

In conclusion, data from experimental models, and to a lesser extent from clinical studies in depression, obesity and psoriatic disease, provide the impetus for the understanding for the complex nature of the immunopathogenesis of these manifestations, and suggest that IL-17 could be a common denominator cross-linking some of involved features at the cellular and molecular level in susceptible individuals. However, these data must be treated with caution, as no clear cut evidence for a beneficial role of anti-IL-17 treatment (other than that related to skin lesions and arthritis) in managing anxiety, depression is currently provided, and data by no means are conclusive. Also, a direct effect of anti-IL17 inhibitors in obesity is not clearly documented and this is rather troubling for those promoting the hypothesis suggesting that anti-IL17 neutralizing antibodies may indeed have a clinical impact in fighting obesity. In the clinical setting, body weight does not appear to influence the efficacious effect of anti-IL17 inhibitors in patients with psoriatic disease and this cannot be underestimated. Circumstantial evidence supporting the opposite effect is also weak, raising the expectation of more well controlled, prospective studies investigating these issues in the near future. Limited data on male patients with PsA, have shown that secukinumab treatment is associated with a decrease in resestin and chemerin (but not adiponectin) at 6 months post-treatment compared to baseline. Such a decrease on adipokines was not noted in women with PsA. It must also be emphasized that, as per anti-TNF-α biologics it is not clear to what extent and under which circumstances anti-IL-17 biologics can cross the blood brain barrier, suggesting that their presumed ability to exert any anti-depressant effect would derive mainly from peripheral inhibition of IL-17 rather than a direct effect of those biologics on the brain (50). Nevertheless, the role played in by the IL-17 axis in the development of depression (and obesity) which are frequently regarded as comorbidities of psoriatic disease needs urgent attention and further exploration, mainly because it will assist efforts to better understand whether immunodysregulation involving IL-17 is indeed involved in the induction of either obesity or depression (or both).

## Author Contributions

EZ and DB had the original idea and scripted considerable part of original draft. AD and VS had performed extensive literature search and scripted part of the manuscript. ED scripted part of the original draft and edited the manuscript. DB prepared the artwork DB and EZ had edited the final version of the manuscript. All authors contributed to the article and approved the submitted version.

## Funding

This research was funded in part by the Special Account for Research Grants University of Thessaly, grant numbers 6357, 5158 & 5847.

## Conflict of Interest

EZ: Lilly, Genesis Pharma, Janssen, LEO Pharma, Novartis, UCB, Pfizer, Sanofi Genzyme, AbbVie—speaker honoraria/or paid investigator. ED: Allergan, Novartis, Genesis, ELPEN, Bayer, Teva, Merck-Serono, Genzyme-Sanofi, Roche, UCB: speaker or chairman horonaria, advisory or travel grants, and clinical research-educational support grants. DB: AbbVie, Novartis, Genesis, ELPEN, Pfizer, Aenorasis, Menarini, Kopper, ITF Hellas, Roche, MSD, GSK, Hospital Line-speaker or chairman horonaria or paid investigator or clinical research and educational support grants.

The remaining authors declare that the research was conducted in the absence of any commercial or financial relationships that could be construed as a potential conflict of interest.
